# Effects of collision cascade density on radiation defect dynamics in 3*C*-SiC

**DOI:** 10.1038/srep44703

**Published:** 2017-03-17

**Authors:** L. B. Bayu Aji, J. B. Wallace, S. O. Kucheyev

**Affiliations:** 1 Lawrence Livermore National Laboratory, Livermore, California 94550, USA; 2 Department of Nuclear Engineering, Texas A&M University, College Station, Texas 77843, USA

## Abstract

Effects of the collision cascade density on radiation damage in SiC remain poorly understood. Here, we study damage buildup and defect interaction dynamics in 3*C*-SiC bombarded at 100 °C with either continuous or pulsed beams of 500 keV Ne, Ar, Kr, or Xe ions. We find that bombardment with heavier ions, which create denser collision cascades, results in a decrease in the dynamic annealing efficiency and an increase in both the amorphization cross-section constant and the time constant of dynamic annealing. The cascade density behavior of these parameters is non-linear and appears to be uncorrelated. These results demonstrate clearly (and quantitatively) an important role of the collision cascade density in dynamic radiation defect processes in 3*C*-SiC.

The understanding of radiation damage phenomena in SiC is desirable for the development of radiation tolerant SiC-based nuclear ceramics and for the control of lattice disorder associated with ion-beam processing of electronic devices^1^,^2^. Despite decades of research, however, our understanding of radiation damage processes in SiC remains limited, particularly in regimes with pronounced dynamic annealing (DA). Such DA refers to the migration and interaction of point defects (during irradiation) after the thermalization of ballistically generated collision cascades for time scales ≳1 ps^3^. (See, for example, a review by Kinchin, G. W. and Pease, R. S. The displacement of atoms in solids by radiation. *Rep. Prog. Phys*. **18**, 1–51 (1955)).

Damage buildup under pronounced DA is strongly influenced by irradiation conditions^3^. For a fixed ion dose (Φ), the type and concentration of radiation-produced stable lattice defects depend on sample temperature (*T*) during irradiation, the dose rate (*F*), ion mass (*m*), and energy (*E*). Here, we focus on the influence of the displacement generation rate (*R*_*gen*_) that is controlled by *m* and *F*. The *F* determines the average frequency of ion impacts onto any given area on the sample surface and, hence, the depth-dependent average rate of the ballistic generation of atomic displacements.

The *R*_*gen*_ depends not only on *F* but also on *m*. Generally, *m* determines the following three aspects of ballistic displacement generation: (i) the shape of the displacement generation depth profile, (ii) the partition between the energy deposited by ions in electronic and nuclear energy loss processes, and (iii) the effective volumetric density of displacements in collision cascades. For relatively low *E* ions used in the present work (with an electronic energy loss ≲1 keV nm^−1^), electronic excitation effects (although poorly understood and requiring further studies) are typically negligible for metals and predominantly covalent ceramics such as SiC^3^,^4^. In this case, the *m* effect is related to the difference in the density of collision (sub)cascades (*ρ*_*cascade*_) generated by different ion species. Lighter ions create relatively dilute cascades characterized by large average distances between adjacent displacements within cascades. Heavier ions create denser cascades. Several algorithms have been developed to calculate average (sub)cascade densities^5^,^6^,^7^. For most materials, experiments have revealed an increase in the efficiency of the formation of stable lattice disorder with increasing *m*^6^,^7^,^8^ (See, for example, a review by Davies, J. A. In: *Ion Implantation and Beam Processing*, edited by Williams, J. S. and Poate, J. M. (Academic, New York, 1984)). This has generally been attributed to either nonlinear energy spike phenomena or more efficient intra-cascade defect clustering^6^,^7^,^8^,^9^,^10^.

Here, we focus on the *R*_*gen*_ effects on damage buildup and DA in 3*C*-SiC, which is the cubic polymorph of SiC (also often referred to as *β*-SiC). The importance of *m* in damage buildup in 3*C*-SiC could be inferred from the analysis of previous measurements of the *T* dependence of the amorphization dose (Φ_*amorph*_)^11^,^12^,^13^,^14^,^15^. The existing Φ_*amorph*_(*T*) data for 3*C*-SiC for 2 MeV electrons^11^ and 1.5 MeV Xe^12^, 360 keV Ar^13^, 1 MeV Kr^14^, and 500 keV Ar ions^15^ was recently summarized in ref. 15. Its analysis, however, reveals an almost random pattern of the dependence of the Φ_*amorph*_ on *m* (for any given *T*), with the highest Φ_*amorph*_ for electrons^11^ and the lowest for 500 keV Ar ions^15^. This apparent inconsistency could be attributed to differences in *F, E*, and the method of defect characterization used by different groups^11^,^12^,^13^,^14^,^15^. Hence, in the present work, we study the *m* effect in 3*C*-SiC with all the other irradiation conditions kept constant or otherwise controlled.

Ion *m* effects on Φ_*amorph*_ have also been studied for 6*H*-SiC and 4*H*-SiC polymorphs^13^,^16^,^17^,^18^,^19^,^20^,^21^. Although an increase in the damage production efficiency with increasing *m* has typically been observed in most previous studies of 6*H*- and 4*H*-SiC^13^,^16^,^17^,^18^,^19^, negligible^20^ and inverse^21^ (i.e., a reduction in the damage production efficiency for heavier ions) *m* effects have also been reported. Extrapolation of these results to the case of 3*C*-SiC is, however, not fully justified, given previous reports of a significant difference in the radiation response of different SiC polymorphs^19^,^22^,^23^. At elevated *T*s, DA processes are expected to be different in various SiC polymorphs since such processes are controlled by the type and properties of lattice-structure-specific point and extended defects^15^.

Damage buildup studies are traditionally performed by bombardment with continuous (rather than pulsed) ion beams^3^. Such experiments provide limited insight into the dynamics of point defect interaction during irradiation. Our recent work^24^,^25^,^26^,^27^,^28^,^29^ has demonstrated that defect interaction dynamics can be accessed in experiments with pulsed ion beams when the Φ is delivered as a train of equal square pulses with a duration of *t*_*on*_ and an instantaneous dose rate of *F*_*on*_, separated by a passive portion of the beam duty cycle of *t*_*off*_. Such pulsed beam experiments allow us to measure the characteristic DA time constant (*τ*) and the defect diffusion length (*L*_*d*_) by studying the dependence of stable lattice disorder on *t*_*off*_ and *t*_*on*_, respectively^24^,^25^,^26^,^27^,^28^,^29^. We have recently measured a *τ* of ~3 ms and a *L*_*d*_ of ~10 nm for 3*C*-SiC bombarded at 100 °C with pulsed beams of 500 keV Ar ions^28^,^29^.

Here, we study both the damage buildup behavior and defect interaction dynamics in 3*C*-SiC bombarded at 100 °C with different *m* ions (from ^20^Ne to ^129^Xe) with a fixed *E* of 500 keV (see Table 1). We focus on the effects of the collision cascade density on radiation dynamics at a fixed *T*. We have chosen 100 °C since this is an irradiation regime with pronounced DA^15^. It is also relevant for elevated-temperature implantation of SiC-based devices and for SiC performance in a nuclear reactor environment^1^,^2^. In this *E* range (0–500 keV), for all four ion species, the electronic energy loss is weakly and non-monotonically dependent on *m*, while the nuclear energy loss experiences a dramatic increase with increasing *m*^30^. Our results reveal that the amorphization cross-section constant (*ξ*_*amorph*_) and *τ* increase and the DA efficiency (*ξ*_*DA*_) decreases with increasing *m*. Ion *m* (and, hence, *ρ*_*cascade*_) dependencies of different parameters are, however, non-linear and appear to be uncorrelated. This clearly demonstrates that the *ρ*_*cascade*_ non-trivially influences not only the efficiency of damage accumulation but also defect interaction dynamics in SiC.

## Results and Discussion

### Damage buildup

**Figure 1:**
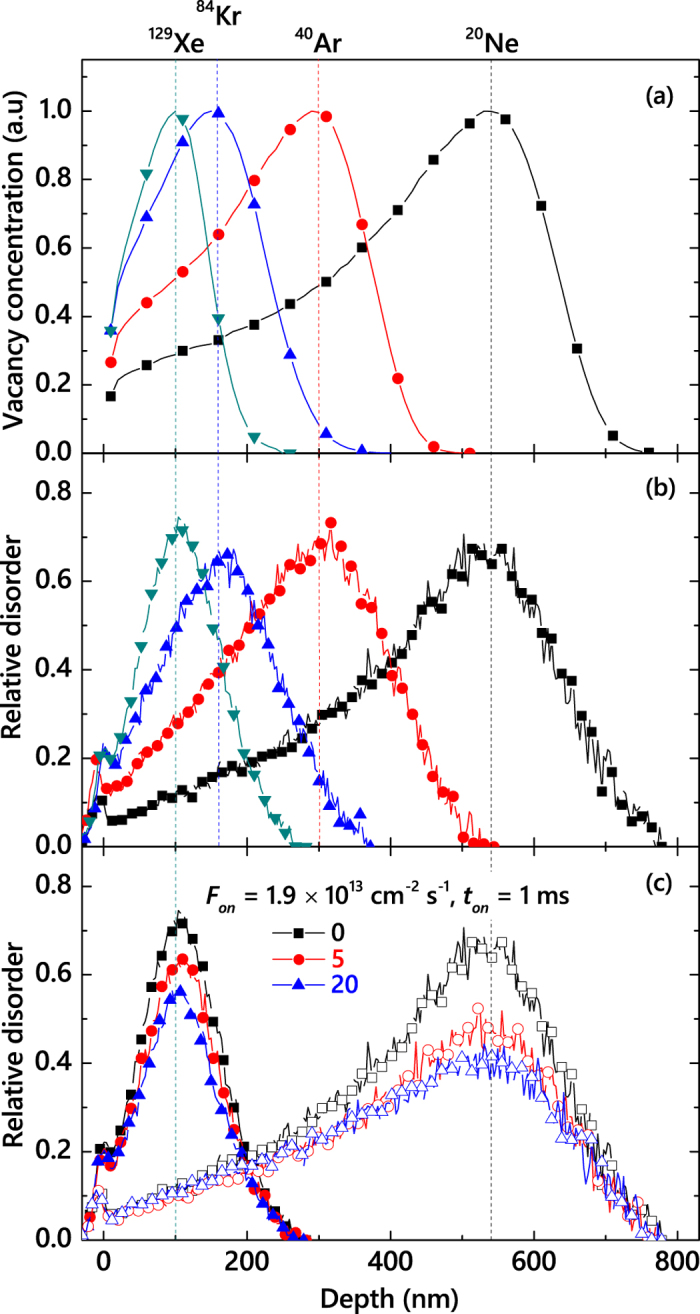
(**a**) Normalized depth profiles of the concentration of lattice vacancies ballistically generated in SiC by irradiation with 500 keV Ne, Ar, Kr, or Xe ions. Positions of the vacancy distribution maxima are indicated by vertical dashed lines. (**b**) Selected depth profiles of relative disorder in 3*C*-SiC irradiated at 100 °C with continuous beams of Ne, Ar, Kr, or Xe ions with a *F* of 1.9 × 10^13^ cm^−2^ s^−1^ to Φs of 0.4, 0.31, 0.33, and 0.32 DPA, respectively. The profile for Ar ions is taken from ref. 15. (**c**) Selected depth profiles of relative disorder in 3*C*-SiC bombarded with pulsed Ne (open symbols) or Xe (closed symbols) ion beams to Φs of 0.4 and 0.32 DPA, respectively, with different values of *t*_*off*_ (indicated in the legend in ms), *t*_*on*_ = 1 ms, and *F*_*on*_ = 1.9 × 10^13^ cm^−2^ s^−1^. For clarity, only every 5th experimental point is depicted in (**b**) and (**c**).

Insight into the physics of radiation damage formation could often be gained by analyzing the damage buildup behavior: the dependence of the amount of stable post-irradiation lattice disorder on Φ. Hence, before embarking on pulsed beam experiments, we first measured the damage buildup under continuous beam irradiation (i.e., *t*_*off*_ = 0 ms). Figure 1(a) shows normalized depth profiles of lattice vacancies ballistically generated in SiC by 500 keV Ne, Ar, Kr, or Xe ions, calculated with the TRIM code^30^. All four profiles show expected unimodal Gaussian-like shapes, with peaks at depths that we refer to as *R*_*pd*_s, indicated by vertical dashed lines in Fig. 1(a) (at 540, 300, 155, and 100 nm). Such *R*_*pd*_s decrease with increasing *m*, which is also expected from ion ballistics^30^.

Representative experimental depth profiles of relative disorder for bombardment with continuous ion beams for these four different ion species and selected Φs are plotted in Fig. 1(b). A comparison of Fig. 1(a) and (b) shows that, for all four ion species, shapes of profiles of stable lattice disorder and ballistically generated vacancies closely resemble each other. The maximum bulk damage is observed at depths close to the corresponding *R*_*pd*_s. The difference is in relatively small surface peaks in Fig. 1(b), reflecting surface disordering and the expected surface scattering of the probing He ions in ion channeling experiments. For all four ion species, bulk damage peaks (with heights of *n*) are centered at depths close to their respective *R*_*pd*_ values in Fig. 1(b). These observations are in agreement with results of our recent systematic study^15^ of the *T*-dependence of damage buildup in 3*C*-SiC under 500 keV Ar ion bombardment.

**Figure 2:**
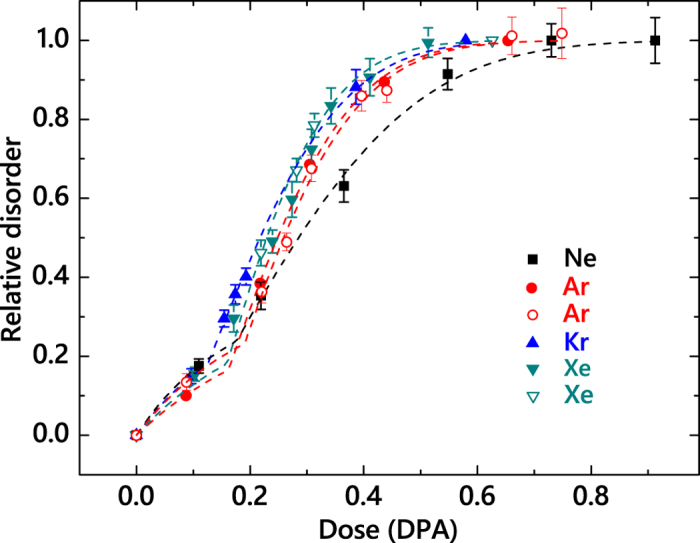
Dose dependencies of relative disorder at the maximum of the bulk defect peak for 3*C*-SiC bombarded at 100 °C with continuous beams of 500 keV Ne, Ar, Kr, or Xe ions (closed symbols) with a *F* of 1.9 × 10^13^ cm^−2^ s^−1^ and (open symbols) with lower *F*s of 4 × 10^12^ cm^−2^ s^−1^ for Xe ions and 8.4 × 10^12^ cm^−2^ s^−1^ for Ar ions. Dashed lines are results of fitting with a stimulated amorphization model from ref. 15.

Figure 2 summarizes bulk damage buildup curves [i.e., *n*(Φ) dependencies] for bombardment with continuous beams of the four ion species. All the damage buildup curves of Fig. 2 are sigmoidal, suggesting nucleation-limited (i.e., stimulated) defect accumulation. Shown in Fig. 2 by dashed lines are results of the fitting of the damage buildup curves with a phenomenological stimulated amorphization model from ref. 15. Within this model, the total damage level is given by the following expressions:


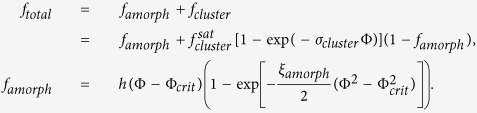


Here, *f*_*amorph*_ is the fraction of atoms in the amorphous phase, *f*_*cluster*_ is the atomic fraction of stable point defect clusters, *σ*_*cluster*_ is the cluster production cross-section, 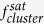 (with 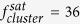
%, based on results from ref. 15) is the maximum saturation fraction of defect clusters in the lattice, *h(x*) is the Heaviside step function [*h(x*) = 0 for *x* < 0 and *h(x*) = 1 for *x* ≥ 0], Φ_*crit*_ is the critical dose above which amorphization proceeds, and *ξ*_*amorph*_ is the amorphization cross-section constant.

**Figure 3:**
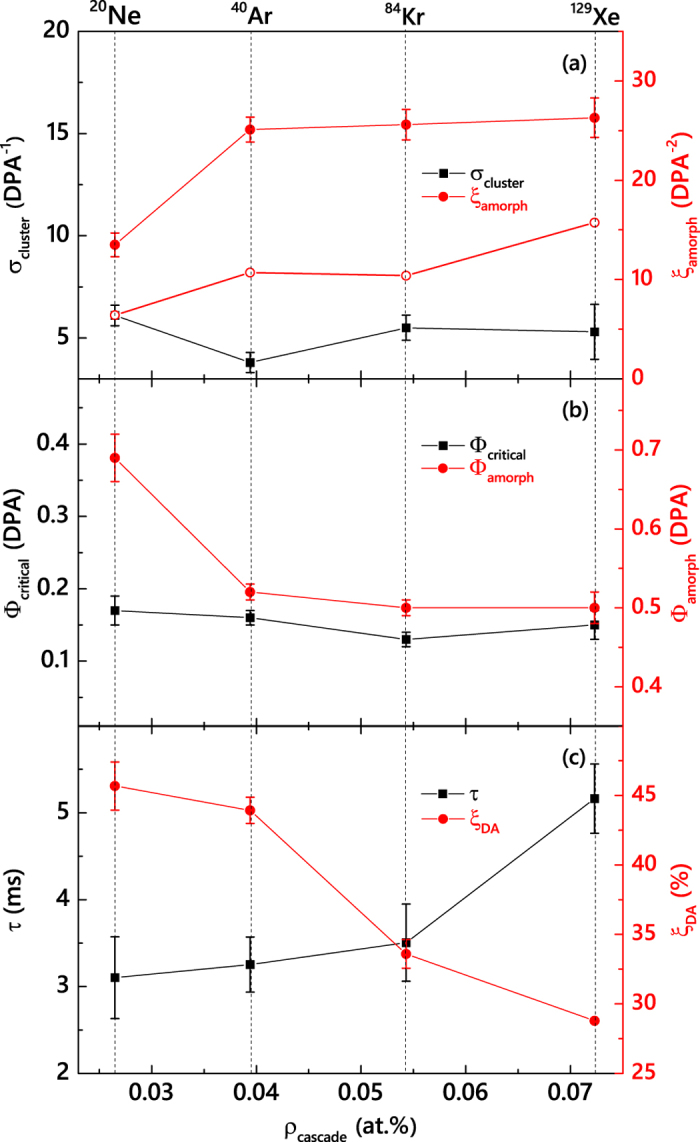
Cascade density (*ρ*_*cascade*_) dependencies of (**a**) the cross-section for the formation of point defect clusters (*σ*_*cluster*_) and the amorphization cross-section constant (*ξ*_*amorph*_); (**b**) the amorphization dose (Φ_*amorph*_) and the critical dose for the onset of amorphization (Φ_*crit*_); and (**c**) the effective time constant of DA (*τ*) and the DA efficiency (*ξ*_*DA*_) for 3*C*-SiC bombarded at 100 °C with 500 keV Ne, Ar, Kr, or Xe ions. Open symbols in (**a**) show *ξ*_*amorph*_ for the case of *F* → 0 obtained by the analysis of pulsed beam data measured with *t*_*off*_ ≫ *τ*.

Figure 2 shows that such a stimulated amorphization model provides an excellent fit for all the cases. Ion *m* (and, hence, *ρ*_*cascade*_) dependencies of the fitting parameters (*σ*_*cluster*_, *ξ*_*amorph*_, and Φ_*crit*_) and the Φ_*amorph*_ (taken as the Φ corresponding to *n* = 0.95) are shown in Fig. 3(a) and (b). It is seen that, within fitting errors, *σ*_*cluster*_ and Φ_*crit*_ are independent of *m*, while *ξ*_*amorph*_ exhibits a monotonic increase with increasing *m*. A fast increase in *ξ*_*amorph*_ occurs on increasing *m* from Ne to Ar, followed by a saturation stage (or a minor growth within error bars) with further increasing *m* from Ar to Xe. For example, for Φs resulting in 0.4 DPA, the bulk damage level differs by ~1.2 times after Ne and Ar ions bombardment, while the corresponding difference in *n* between cases of Ar and Xe ions is only ~8%.

The difference in damage buildup revealed by Figs 2, 3(a) and (b) could be related not only to different *ρ*_*cascade*_s created by different ion species but also to different *R*_*gen*_s. Indeed, the four damage buildup curves shown by solid symbols in Fig. 2 were measured with a constant *F* of 1.9 × 10^13^ cm^−2^ s^−1^. Since heavier ions create more atomic displacements per ion at their *R*_*pds*_, they also result in larger *R*_*gen*_s when a constant *F* is maintained for different *m*. For example, irradiation with Xe ions results in an ~5 times larger *R*_*gen*_ than for the case of Ar ion bombardment with the same *F* (see Table 1).

Hence, in order to differentiate between *ρ*_*cascade*_ and *R*_*gen*_ effects (i.e., between intra-cascade and inter-cascade phenomena, related to the average dose rate), we have measured damage buildup curves for Ar and Xe ion bombardment with lower *F*s of 8.4 × 10^12^ and 4 × 10^12^ cm^−2^ s^−1^, respectively. Such lower *F* Xe ion bombardment results in the same *R*_*gen*_ (at *R*_*pd*_) as that for Ar ions with a *F* of 1.9 × 10^13^ cm^−2^ s^−1^, while lower *F* Ar ion bombardment was done in order to match the *R*_*gen*_ for Ne ion bombardment (see the *R*_*gen*_ column of Table 1). Results are shown in Fig. 2 by open symbols, demonstrating a negligible dose-rate effect for both Ar and Xe ion bombardment for the *F* range studied.

**Figure 4:**
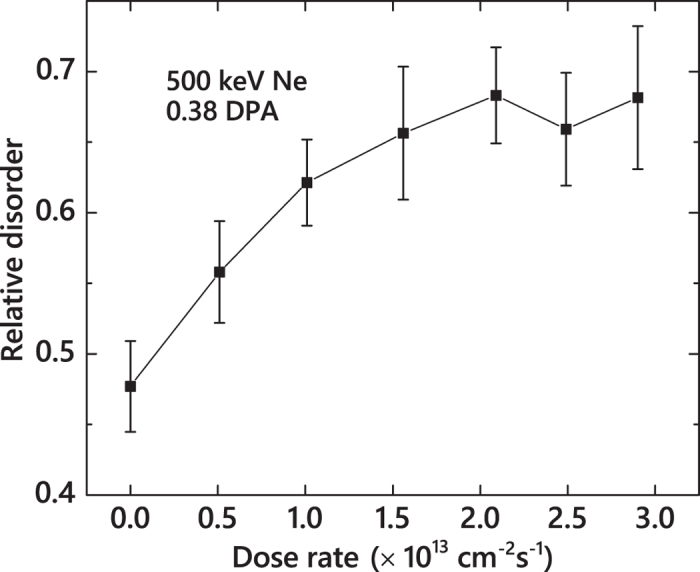
Dose-rate dependence of relative disorder at the maximum of the bulk defect peak for 3*C*-SiC bombarded at 100 °C with a continuous beam of 500 keV Ne ions to a Φ of 1 × 10^15^ cm^−2^, corresponding to 0.38 DPA. The data point for *F* → 0 was obtained for irradiation with a pulsed beam with a *t*_*off*_ of 50 ms.

We further clarify that the difference in damage buildup between Ne and the heavier ions is not related to a lower *R*_*gen*_ of Ne ions. For Ne ion bombardment, a very large *F* of ~1.8 × 10^14^ cm^−2^ s^−1^ would be required in order to match the *R*_*gen*_ (at *R*_*pd*_) of Xe ions with a *F* of 1.9 × 10^13^ cm^−2^ s^−1^ (see Table 1). Such large current beams are beyond our experimental capabilities. Instead, we have measured the *n(F*) dependence for Ne ions, as shown in Fig. 4. It is seen that *n* sub-linearly increases with *F* and saturates for *F* ≳ 1.5 × 10^13^ cm^−2^ s^−1^. This suggests a negligible contribution of the effect of the average dose rate to a pronounced difference in *ξ*_*amorph*_ between Ne and heavier ions. The difference observed in the damage buildup of Ne and heavier ions is, therefore, attributed to *ρ*_*cascade*_ phenomena. This conclusion is further supported by open symbols in Fig. 3(a) showing the *ρ*_*cascade*_ dependence of *ξ*_*amorph*_ for the case of *F* → 0 obtained by the analysis of pulsed beam data measured with a *t*_*off*_ ≫ *τ*, as discussed below.

It is interesting to compare *ρ*_*cascade*_ effects revealed by Figs 2, 3(a) and (b) for 3*C*-SiC with those in other non-metallic materials. Previous experiments for different materials have revealed that the efficiency of the formation of stable damage generally increases with increasing *m*^6^,^8^,^9^,^10^,^13^,^16^,^17^,^18^,^19^,^31^,^32^. However, even the qualitative behavior is non-trivial and depends strongly on the material and irradiation conditions such as Φ, *F*, and *T*. For example, for Si at room *T*, an increase in the *ρ*_*cascade*_ leads to a gradual increase in the damage formation efficiency^10^. For GaN at room *T*, there is a threshold-like increase in damage with increasing *m*, suggesting an important role of energy spikes^6^,^9^. For ZnO, the bulk disorder is essentially independent of the *ρ*_*cascade*_, while the evolution of near-surface damage exhibits strong and complex *ρ*_*cascade*_ effects^31^,^32^. As already mentioned, an increase in the damage production efficiency with increasing *m* has also been observed in most previous studies of 6*H* and 4*H* polymorphs of SiC^13^,^16^,^17^,^18^,^19^, although we are not aware of any previous systematic studies of the *m* effect in 6*H*- and 3*C*-SiC at room *T* and above when DA processes are pronounced. The physical mechanisms behind such differences between *ρ*_*cascade*_ effects in different materials are currently not well understood and deserve further systematic studies.

### Defect interaction dynamics

Based on the above damage buildup data (Fig. 2), we have chosen Φs for pulsed beam experiments so that, for *t*_*off*_ = 0 (i.e., continuous beam irradiation), *n* is in the range of 0.6–0.8, which is in a nonlinear regime of damage buildup with pronounced DA^15^. We have measured *n(t*_*off*_) dependencies for all four ion species in order to obtain *τ* values. Figure 1(c) shows two sets of representative depth profiles of relative disorder in 3*C*-SiC bombarded with Ne or Xe ions with three different *t*_*off*_ values (given in the legend) and all the other parameters kept constant. It is seen that, for both Ne and Xe cases, *n* decreases with increasing *t*_*off*_, while the damage level at the sample surface remains unchanged, suggesting different dynamic mechanisms of bulk and surface disordering. This behavior is qualitatively similar to that previously reported in pulsed-beam studies of Si bombarded at room *T* with 500 keV Ne, Ar, Kr, or Xe ions, of 3*C*-SiC irradiated at 100 °C with 500 keV Ar ions, and of 4*H*-SiC bombarded with 500 keV Ar ions in the *T* range of 25–250 °C^26^,^27^,^28^.

**Figure 5:**
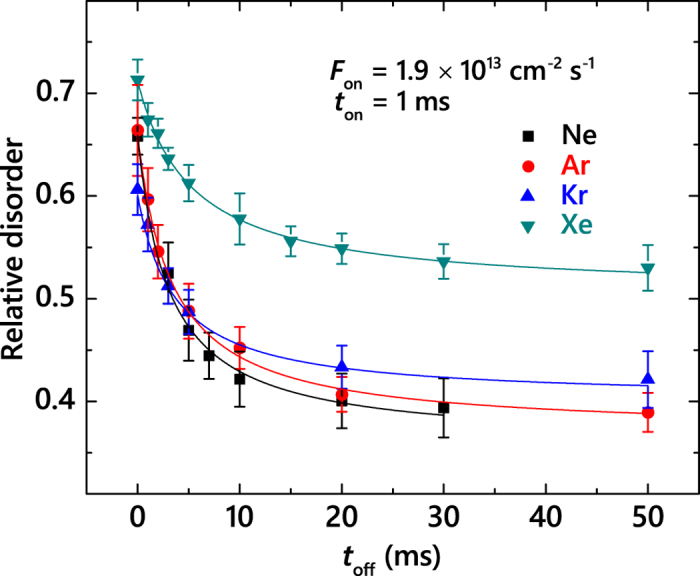
Level of relative bulk disorder in 3*C*-SiC bombarded at 100 °C with pulsed beams of 500 keV Ne, Ar, Kr, and Xe ions with *t*_*on*_ = 1 ms and F_on_ = 1.9 × 10^13^ cm^−2^ s^−1^ as a function of t_off_ to Φs of 0.4, 0.31, 0.33, and 0.32 DPA, respectively. Data for Ar ions is from ref. 28. Fitting curves of the data with the second order decay equation are shown by solid lines.

We have found such a reduction in *n* with increasing *t*_*off*_ for 3*C*-SiC for all four ion species (Fig. 5). Solid lines in Fig. 5 are fits of the data via the Marquardt-Levenberg algorithm^33^ with the second order decay equation 
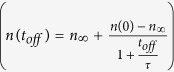
. Here, *n*_∞_ is relative disorder for *t*_*off*_ ≫ *τ*. All the *n(t*_*off*_) dependencies from Fig. 5 obey the second order decay better than the first order (i.e., exponential) decay.

The effect of *m* (and, hence, *ρ*_*cascade*_) on *τ* is summarized in Fig. 3(c), revealing a monotonic increase in *τ* with increasing *m*. Such an increase in *τ* with increasing *m* is consistent with our recent finding for Si at room *T*^26^. Slower defect relaxation dynamics for irradiation with heavier ions could suggest that heavier ions result in the formation of lattice defects that act as efficient traps for migrating point defects, slowing down the defect relaxation dynamics. Alternatively, an increase in *τ* with increasing *m* could reflect a possible dependence of *τ* on the the instantaneous concentration of mobile (rather than stable) defects at the end of each pulse^26^. Future systematic studies of *τ(t*_*on*_) dependencies should shed light on the physics behind the *ρ*_*cascade*_ dependence of *τ* revealed here.

Also plotted in Fig. 3(c) is the *m* dependence of the DA efficiency (*ξ*_*DA*_): 
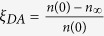
. As discussed in detail previously^26^, for our choice of pulsing parameters, *ξ*_*DA*_ is the magnitude of the dose-rate effect; i.e., the difference between *n* for continuous beam irradiation with dose rates of *F* = *F*_*on*_ and *F* → 0. Figure 3(c) shows that, with increasing *m, ξ*_*DA*_ gradually decreases from ~46 to ~29%.

The above results clearly demonstrate the complexity of DA processes in 3*C*-SiC, revealing non-linear and uncorrelated *ρ*_*cascade*_ dependencies of the *ξ*_*amorph*_ and defect dynamics parameters (*τ* and *ξ*_*DA*_). A comparison of data from Figs 2, 3(a) and (c) also reveals another unexpected result: a strong *ρ*_*cascade*_ dependence of the dose-rate effect measured in pulsed beam experiments [*ξ*_*DA*_ in Fig. 3(c)] and minor dose-rate (i.e., *R*_*gen*_) effects studied directly by measuring damage buildup for different ion species and *F* values [Figs 2 and 3(a)]. This observation suggests that *n(F*) dependencies for different *m* ion have shapes similar to that for the case of 500 keV Ne ion bombardment shown in Fig. 4, with a pronounced saturation effect for large *F* values. With such non-linear *n(F*) dependencies, the difference between the magnitudes of the dose-rate effect measured by the pulsed beam method and traditional dose-rate dependent damage buildup is related to the difference in the range of *F* values over which these measurements are done. In the pulsed beam method, measurements extend to *F* → 0, where the dose-rate effect (i.e., the slope of the *n(F*) dependence) is maximum. These observations warrant future systematic dose-rate studies in 3*C*-SiC.

In summary, we have studied the damage buildup and defect interaction dynamics in 3*C*-SiC bombarded at 100 °C with 500 keV Ne, Ar, Kr, or Xe ions. We have found that, with increasing *ρ*_*cascade*_ by increasing ion *m* from Ne to Xe, the *ξ*_*DA*_ (i.e., the magnitude of the dose-rate effect) gradually decreases, accompanied by an increase in the *τ* from 3.1 to 5.2 ms. At the same time, the *ξ*_*amorph*_ increase significantly only on changing ion *m* from ^20^Ne to ^40^Ar, with a weak *m* dependence with a further increase in ion *m*. These results clearly demonstrate that radiation defect dynamics in 3*C*-SiC is complex and strongly depends on the collision cascade density. These observations have important implications for the development of predictive modeling capabilities to describe radiation damage processes in SiC.

## Methods

Depth profiles and three-dimensional distributions of ballistically-generated lattice vacancies were calculated with the TRIM code (version SRIM-2013.00, full cascade calculations)^30^ with an atomic concentration of SiC of 9.64 × 10^22^ atoms cm^−3^ (ref. 2) and threshold energies for atomic displacements of 20 and 35 eV for C and Si sublattices, respectively^34^. To convert to displacements per atom (DPA) at the *R*_*pd*_, Φs in 10^14^ cm^−2^ are multiplied by 0.036, 0.085, 0.193, and 0.342 for Ne, Ar, Kr, and Xe ions, respectively.

Cascade densities (*ρ*_*cascade*_s) at *R*_*pd*_s were calculated based on the algorithm similar to that proposed by Heinisch and Singh^5^. We define the *ρ*_*cascade*_ as the average local density of lattice vacancies within individual cascades with an averaging radius of 10 nm. Such an averaging radius was chosen to be comparable with our recent estimates of the *L*_*d*_ in 3*C*-SiC^29^. Values of *ρ*_*cascade*_ were obtained by averaging over ≳600 individual cascades.

We used single-crystal epilayers of (001) 3*C*-SiC (on Si wafers with a diameter of 100 mm) obtained from NOVASiC. The epilayers had a thickness of ≳2 *μ*m and a resistivity of 1–10 Ω cm. The crystal quality of as-received films was verified by measuring a minimum 2 MeV ^4^He ion channeling yield of ~1.5%, consistent across the wafer. A transmission electron microscopy study of as-received SiC films was reported in ref. 15.

The 4 MV ion accelerator (National Electrostatics Corporation, model 4UH) at Lawrence Livermore National Laboratory was used for both ion irradiation and ion beam analysis. The ion irradiation conditions used in this study are summarized in Table 1. Ion bombardment was done with 500 keV ^20^Ne^+^, ^40^Ar^+^, ^84^Kr^+^, or ^129^Xe^+^ ions at ~7° off the [100] direction to minimize channeling effects. All irradiations were performed in the broad beam (rather than raster) mode^24^. To improve thermal contact, the samples were attached to the Cu sample holder with conductive Ag paste. The target *T* was kept at 100 ± 1 °C. Irradiated areas were ~4 × 5 mm^2^. Total Φs were in the range of 0.1–0.9 DPA. Ion beam pulsing was achieved by applying high voltage pulses to a pair of parallel plates to deflect the ion beam off the target so that the total Φ was split into a train of equal square pulses with a dose per pulse of Φ_*pulse*_ = *F*_*on*_*t*_*on*_. The adjacent pulses were separated by time *t*_*off*_.

In such pulsed beam experiments, it is important to maintain a constant *F*_*on*_ throughout the experiment and have good control of both Φ and *T*. In our experiments, all the data points in each *n(t*_*off*_) curve were measured in the same experimental run to avoid possible slight variations in dosimetry or wafer *T* between different runs. Control of the dosimetry and *T* is crucial since we are dealing with a steep portion of the damage buildup curve (Fig. 2) and an exponential *T* dependence of the damage formation efficiency^15^.

Depth profiles of lattice disorder in the Si sublattice were measured *ex-situ* at room *T* with 2 MeV ^4^He ions incident along the [001] direction and backscattered into a detector at 164° relative to the incident beam direction. Spectra were analyzed with one of the conventional algorithms^35^ for extracting the effective number of scattering centers (referred to below as “relative disorder”). Values of averaged bulk disorder (*n*, with *n* = 1 corresponding to full amorphization) were obtained by averaging depth profiles of relative disorder over 10 channels (~25 nm) centered on the bulk damage peak maximum (i.e., *R*_*pd*_). Error bars of *n* are standard deviations. For each ion *m, τ* was measured by studying the dependence of *n* on *t*_*off*_, which was varied between 1 and 50 ms, with a constant *F*_*on*_ of (1.9 ± 0.1) × 10^13^ cm^−2^ s^−1^ and a *t*_*on*_ of 1 ms.

## Additional Information

**How to cite this article:** Aji, L. B. B. *et al*. Effects of collision cascade density on radiation defect dynamics in 3*C*-SiC. *Sci. Rep.* **7**, 44703; doi: 10.1038/srep44703 (2017).

**Publisher's note:** Springer Nature remains neutral with regard to jurisdictional claims in published maps and institutional affiliations.

## Figures and Tables

**Table 1:** 

Ion	*F*_*on*_	Φ	*Rgen*	*τ*	*ξamorph*	*ξDA*
	(10^13^ cm^−2^ s^−1^)	(DPA)	(DPA s^−1^)	(ms)	(DPA^−2^)	(%)
^20^Ne	1.9	0.1–0.7	0.007	3.1 ± 0.5	13.5 ± 1.2	45.6 ± 1.7
^40^Ar	1.9	0.1–0.9	0.014	3.2 ± 0.3	25.1 ± 1.3	43.9 ± 0.9
^40^Ar	0.8	0.1–0.8	0.007		24.3 ± 1.8	
^84^Kr	1.9	0.1–0.6	0.037	3.5 ± 0.4	25.6 ± 1.5	33.6 ± 1.1
^129^Xe	1.9	0.1–0.5	0.065	5.2 ± 0.3	26.3 ± 1.9	28.8 ± 0.1
^129^Xe	0.4	0.2–0.7	0.014		25.5 ± 2.9	

Irradiation conditions of this study: the instantaneous dose rate (*F*_*on*_), the total ion dose (Φ), and the displacement generation rate (*R*_*gen*_). Irradiation was performed at 100 °C with an ion energy of 500 keV for all ion masses. Also given are experimental values of the DA time constant (*τ*), the amorphization cross-section constant (*ξ*_*amorph*_), and the DA efficiency (*ξ*_*DA*_).
